# Tracking Child Language Development With Neural Network Language Models

**DOI:** 10.3389/fpsyg.2021.674402

**Published:** 2021-07-08

**Authors:** Kenji Sagae

**Affiliations:** Department of Linguistics, University of California, Davis, Davis, CA, United States

**Keywords:** child language assessment, natural language processing, computational linguistics, language model, IPSyn, neural network

## Abstract

Recent work on the application of neural networks to language modeling has shown that models based on certain neural architectures can capture syntactic information from utterances and sentences even when not given an explicitly syntactic objective. We examine whether a fully data-driven model of language development that uses a recurrent neural network encoder for utterances can track how child language utterances change over the course of language development in a way that is comparable to what is achieved using established language assessment metrics that use language-specific information carefully designed by experts. Given only transcripts of child language utterances from the CHILDES Database and no pre-specified information about language, our model captures not just the structural characteristics of child language utterances, but how these structures reflect language development over time. We establish an evaluation methodology with which we can examine how well our model tracks language development compared to three known approaches: Mean Length of Utterance, the Developmental Sentence Score, and the Index of Productive Syntax. We discuss the applicability of our model to data-driven assessment of child language development, including how a fully data-driven approach supports the possibility of increased research in multilingual and cross-lingual issues.

## Introduction

Measuring the level of syntactic development in child language precisely is useful both in language research and in clinical settings. Although several metrics have been proposed to quantify progress in language development, such as the Index of Productive Syntax (IPSyn; Scarborough, 1990), the Developmental Sentence Score (DSS; Lee and Canter, 1971), and the Language Assessment, Remediation and Screening Procedure (LARSP; Fletcher and Garman, 1988) the most widely used metric remains the Mean Length of Utterance (MLU; Brown, 1973). Although less detailed than many available alternatives, MLU is simple and fast to compute consistently, while metrics based on identification of specific language structures have traditionally required expert manual analysis. Additionally, MLU use in many languages other than English is considerably more straightforward than adaptation of metrics that rely on identification of specific lexical or grammatical items, and MLU is less susceptible to issues relating to differences among varieties of the same language. While there may seem to be inherent trade-offs associated with the use of approaches to tracking language development based on detailed language-specific structural analysis and based on superficial utterance characteristics, we investigate whether accurate measurements of language development can be made quickly, reliably and without reliance on analyses requiring linguistic expertise. Specifically, through the use of data and neural network approaches to natural language processing, we aim to track language development in a way that is as fine-grained as can be obtained with carefully crafted language-specific metrics, but as fast, reliable and widely applicable as with MLU. Our present goal is not to create a new metric, but to examine whether computational models built only from transcribed utterances can capture how child language utterances change through the course of language development at a fine enough resolution to serve as a foundation for new ways to measure syntactic development.

With the development of computational models for syntactic analysis of child language utterances (Sagae et al., 2004, 2010), automatic accurate computation of syntax-based metrics of language development became possible. Identifying the Index of Productive Syntax (IPSyn; Scarborough, 1990) as a measurement tool that has been used widely in research but requires a substantial amount of manual analysis, Sagae et al. (2005) proposed mapping the language structures targeted in IPSyn computation to patterns in parse trees generated by an automatic parser, eliminating manual effort from the process of calculating IPSyn scores. This work provided initial evidence that automatic IPSyn scoring was possible, and served as the basis for subsequent work to make the concept practical, for example through CLAN-IPSyn (accessible at http://talkbank.org). These efforts have highlighted both the promise of more widespread and consistent assessment of syntactic development and the difficulty in matching the quality of analyses produced by experts (MacWhinney et al., 2020; Roberts et al., 2020).

Scoring schemes originally intended for manual computation, such as IPSyn, are designed partly to account for the strengths and limitations of human annotators, without regard for how to leverage syntactic analysis technology. Recognizing the different strengths in manual and automatic syntactic analysis, Lubetich and Sagae (2014) examined the extent to which IPSyn-like scoring can be performed automatically without a pre-defined list of targeted syntactic structures, leaving it up to a data-driven model to select the relevant structures in the output of an automatic syntactic parser. Their approach is to teach a machine to reproduce IPSyn scores just by looking at automatically generated parse trees, with no information about how IPSyn scores are computed or what they mean. Starting from the assumption that these parse trees contain sufficient syntactic information to assess language development, figuring out what structures to focus on is left to the machine.

The ability demonstrated by this approach to produce scores that track language development almost as accurately as with IPSyn, but without the expertise that went into the design of IPSyn, raises the important question of whether computational models of language can learn to measure syntactic development in children from only language data, without any given knowledge about the language acquisition process. This question is not whether a computational model can perform the steps necessary for IPSyn scoring, as in the work of Sagae et al. (2005), or whether a computational model can learn IPSyn scoring from examples, as in the work of Lubetich and Sagae, but whether a computational model derived from child language samples alone can encode its own metric that tracks language development over time as accurately as, or even more accurately than an expertly designed metric like IPSyn. In other words, if the goal is not to model an existing language development metric, but to model the language itself and how it changes over time in individual children, will the resulting model encode a usable language development metric? We investigate this question by creating such a model using neural networks. We base our approach on language modeling using a type of recurrent neural network, but unlike typical language models used in natural language processing that are trained to predict tokens in a string, we additionally have our model sort child language samples chronologically during training. This sorting consists of scoring different language samples produced at different times such that the score for the sample produced later is higher than the score for the sample produced earlier. This is intended to require the model to learn how utterances produced at different stages of development differ. Once the model is trained, it can be used to score a language sample, in the same way one would use existing metrics like IPSyn or MLU. Unlike previous work on automated assessment of child language development, this process does not use a syntactic parser or any information about how to measure language development, such as existing metrics. Although we focus on English, our approach, which requires only transcribed utterances, shares with MLU the advantage of not relying on language-specific resources or language-specific expertise, while having a substantially greater resolution, comparable to that achieved with IPSyn. Using North American English data from the CHILDES database (MacWhinney, 2000), we show that our neural language model successfully discovers how to score child language samples according to language development more accurately than existing implementations of MLU and automated IPSyn scoring. This result suggests that neural network language models are capable of encoding how syntactic development in progresses in English-speaking children, and creates promising directions for accurate data-driven measurement of language development.

## Materials and Methods

Our experiments involve a specific kind of language model based on a type of recurrent neural network, more specifically the Long Short-Term Memory network, or LSTM (Hochreiter and Schmidhuber, 1997). The model is trained using longitudinal child language data from the CHILDES Database. We first describe the neural network model, and present details about the data used. We then describe how the model was trained and how our experiments were conducted.

### Background: Recurrent Language Modeling

Our approach assumes an LSTM language model (Sundermeyer et al., 2015), which is a kind of recurrent neural network language model. This kind of neural language model has been applied successfully in various settings in natural language processing. We provide here only a brief overview of recurrent neural language models to facilitate discussion of our neural network model for language development. For a detail description of LSTM language models, see Sundermeyer et al. (2015).

The general language model formulation commonly assumed in natural language processing and computational linguistics is based on word predictions. Specifically, the model is designed to estimate the probability of strings in the language as the product of the conditional probabilities of each word in the string given the preceding words. Essentially, the model predicts words in a string (a sentence or an utterance) from previous words:

$$\begin{array}{l} P\left(S\right)=P\left(t_{1}t_{2}\ldots t_{N}\right)=\overset{N}{\underset{i=1}{\prod }}P\left(t_{i}|t_{0}\ldots t_{i-1}\right) \end{array}$$

Here, the probability of the string *P*(*S*) is the probability of the word sequence (or *token* sequence) *t*_1_*t*_2_…*t*_*N*_ of length *N*−1, to which special tokens representing the beginning of sentence (BOS) and end of sentence (EOS) have been prepended and appended, respectively, making *t*_0_ BOS, and *t*_*N*_ EOS. The probability of this sequence is the product of the probability of each word *t*_*i*_ given the preceding words *t*_0_…*t*_*i*−1_. Notice that the product above does not include the probability of *t*_0_; since, by how we defined our strings, every string starts with the special token BOS, its probability is 1 and does not affect the product. The probability of the special token EOS, on the other hand, is the probability of ending the string (i.e., ending the utterance or sentence) given all the previous words.

**Figure 1:**
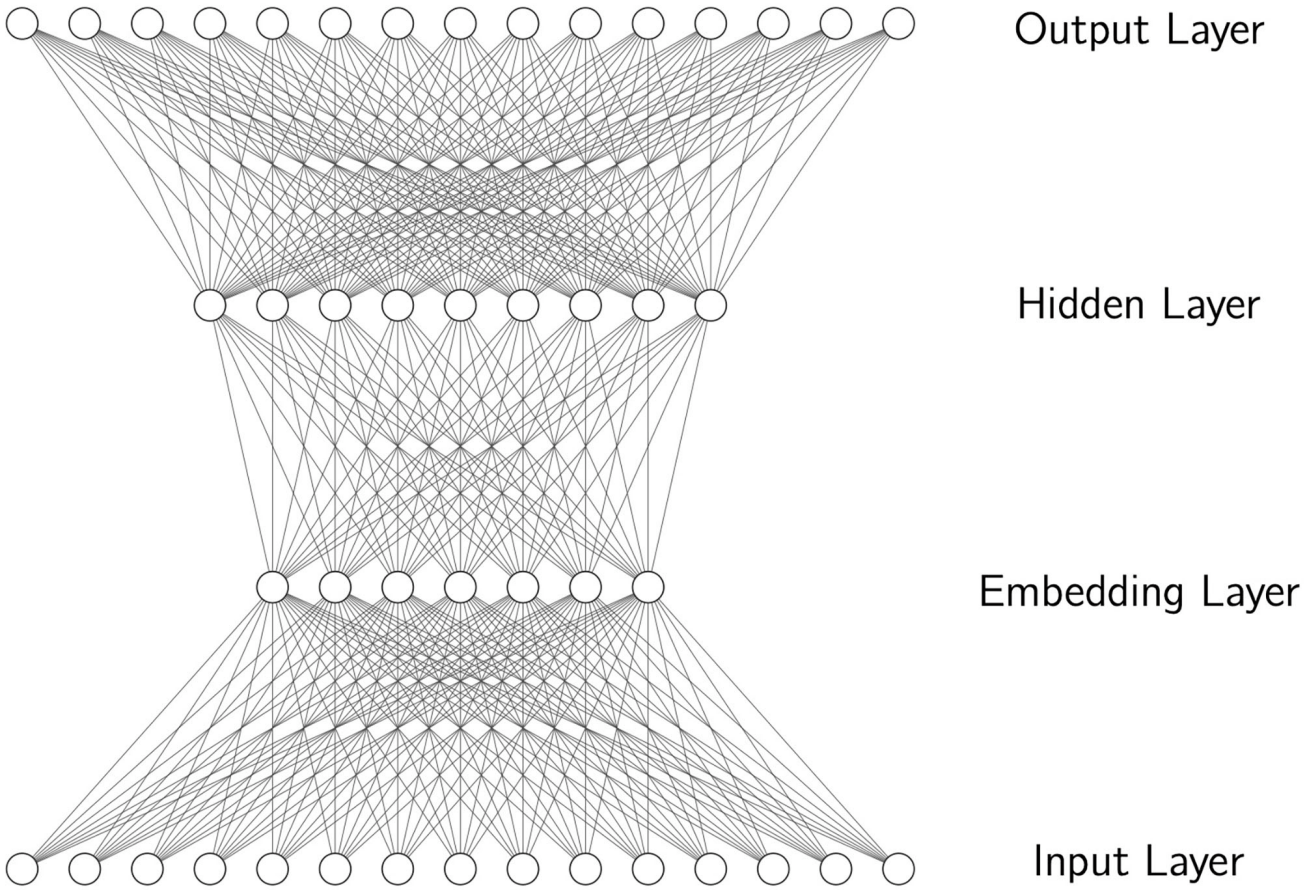
A simple feedforward neural network with an input layer, an embedding layer, a hidden layer, and an output layer. This network can implement a bigram language model with units in the input and output layers representing different words in a vocabulary. When a word is active in the input layer, the unit with highest activation in the output layer is the model's prediction for the next word.

In a language model implemented using neural networks, or a neural language model, these word predictions are made based on spreading activation according to parameters of the neural network. In perhaps its simplest form, where the sequence *t*_0_…*t*_*i*−1_ is approximated according to a first-order Markov assumption as simply *t*_*i*−1_, resulting in a kind of model known as the bigram model, a simple feedforward network takes *t*_*i*−1_ as input and produces *t*_*i*_ as output, as illustrated in Figure 1, where the token *t*_*i*−1_ is represented by a value of 1 in a specific node in the input layer, while the other nodes have value zero, and the output is the node with highest value in the output layer. Notice that the network is made of units organized in layers, and the input word corresponds to a single unit in the input layer. Activation from the input layer spreads to the first hidden layer (the embedding layer), and from there to the second hidden layer, and from there to the output layer, where the unit with highest activation is chosen as the network's prediction. The first hidden layer is often referred to as the embedding layer, and in a trained neural language models it is known to encode meaningful representations of words. Although only two hidden layers are shown (including the embedding layer), the use of more hidden layers is common. In a feedforward network, activation spreads in one direction, from input to output. A unit's activation is a function of the sum of the incoming activation for that unit. The parameters that the model learns from data are the weights that are applied to the connections between units of the network. Typically, the parameters of this kind of network are initialized randomly. Among other things, this means that each word in the vocabulary of the model is initialized to be represented by a random embedding. Over the course of training, where weights are adjusted gradually to increase the probability of predicting the correct output word, the weights learned in the embedding and hidden layers have been found to encode representations of the input and the task that improve prediction of the output. For example, word representations in the embedding layer form a meaningful multidimension space that encodes semantic and syntactic relationships among words (Turian et al., 2010; Mikolov et al., 2013). Intuitively, when learning to predict what word follows *chairs*, the network learns that *chairs* is the plural of *chair*, that *chairs* is related to *seat*, etc.

**Figure 2:**
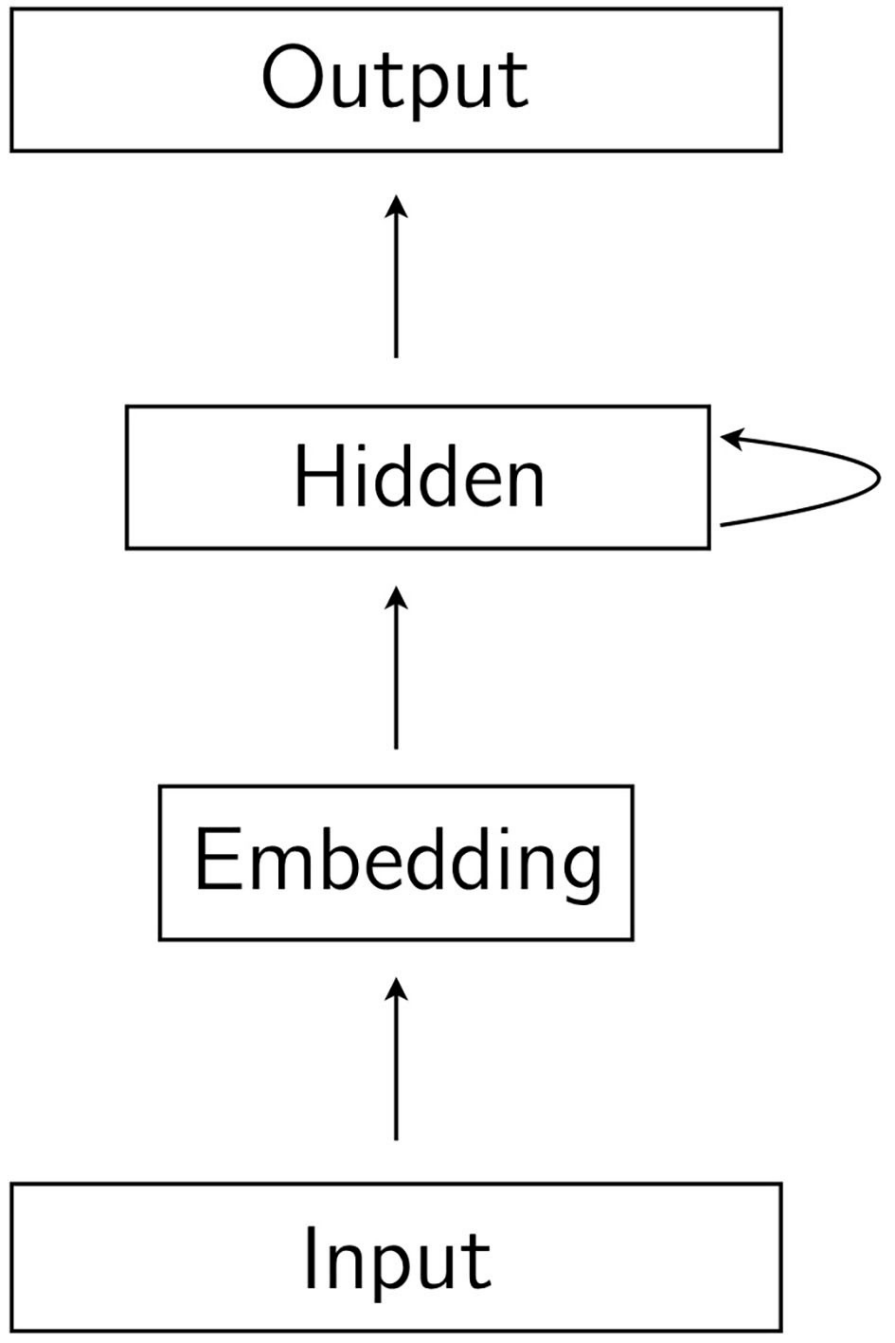
A simple recurrent neural network. The hidden layer receives activation from the embedding layer and from the hidden layer in the previous time step.

**Figure 3:**
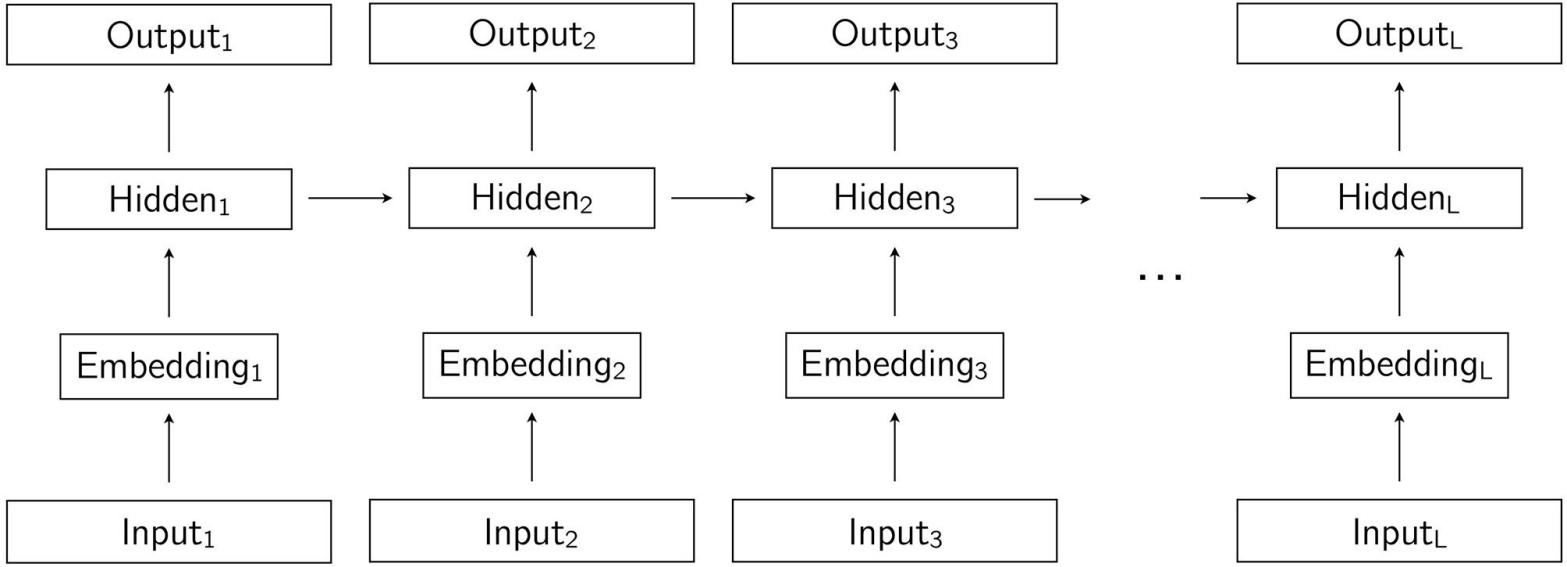
A simple recurrent neural network unrolled for *L* time steps, where *L* is the length of the input string.

In a recurrent neural network, the input is a sequence, and each symbol in the sequence is presented to the network one at a time in consecutive time-steps. In the first time-step, the first symbol is presented, in the second time-step, the second symbol is presented, and so on. In a recurrent neural language model, the input sequence is the string, and the symbols that make up the string are the words, or tokens. The intuitive difference between the feedforward network described in the previous paragraph and a recurrent network is that hidden units in a recurrent network receive activation not just from lower layers, but also from hidden units in the previous time-step. In recurrent language models, the hidden layers are recurrent, with the exception of the embedding layer, which is not recurrent. The term hidden layer is then understood not to include the embedding layer, which is commonly referred to as simply the embedding layer. The result of the recurrence in the network is that, as the string is processed word by word one step at a time, the hidden representation from which the output prediction for word *t*_*i*_ is made is influenced by its preceding words *t*_0_…*t*_*i*−1_. A simple recurrent network is illustrated in Figure 2. In Figure 3, we show the same network unrolled in L time steps, where L is the length of the input sequence. LSTM language models are recurrent language models designed to address specific shortcomings of simple recurrent neural networks. A discussion of these shortcomings and the way in which LSTMs address them are beyond the scope of this brief overview of recurrent neural language models, but are discussed in detail by Goldberg (2017, chapter 15).

One insight about recurrent language models, such as LSTM language models, that is important in understanding our neural network model of child language development is that models with enough hidden units trained with enough data have been found to encode syntactic structure in their hidden representations (Futrell and Levy, 2019; Linzen and Baroni, 2021). Just as the word embedding that result from training neural language models come to encode detailed word representations over the course of training with large corpora because the network learns that such representations are useful in the next-word prediction task, the representations in the hidden layers of a recurrent language model encode syntactic structure because ultimately syntax is important in the next-word prediction task. Intuitively, knowledge of the grammar of the language is necessary to complete or continue sentences. Given enough data and enough parameters, a recurrent language model trained using backpropagation discovers and encodes syntactic information about the language in its hidden layers. Although the syntactic information encoded by neural language models is not always represented in a way that is readily understandable, text generated randomly from large neural language models is surprisingly grammatical and complex, confirming that these models must capture the syntax of the language. Additionally, these language models have been found to be directly useful in tasks explicitly about syntactic structure (Kiperwasser and Goldberg, 2016). Decoding the syntactic and other structure information encoded in neural language models in the context of our current understanding of linguistics is currently a topic of active research (Linzen et al., 2016; Futrell and Levy, 2019; McCoy et al., 2020; Linzen and Baroni, 2021).

### A Neural Network Model of Child Language Development

Our model of child language development is based on the simple assumptions that language is acquired over time and development is monotonic. It is intended to pick up on what changes in utterances through language development, and not to reflect cognitive mechanisms. Monotonicity here does not mean that the child's language is always increasingly more similar to some ultimate form, and it does not mean that development progresses linearly, but simply that typical development does not regress. In other words, the assumption is that given two appropriately sized language samples (lists of utterances) from the same child collected at different times during language development, it should be possible for a model to distinguish between the earlier and later samples. The key idea is that if a model can sort these language samples chronologically, it must do so by figuring out what changes in the language over time. Since recurrent neural language models encode some information about syntax, they are a promising way to encode the language samples to be compared and sorted. Importantly, the goal is to have one model that makes accurate predictions across different children. Even though some children may learn certain things at different rates and at different ages, the model must be able to sort the language samples for a new individual child it has never encountered before. Although the idea of ordering language samples is the key for how we intend to capture changes in language over time, the model is ultimately intended to score individual language samples, in the same way one would score a language sample using an instrument such as IPSyn. We design our model to score individual language samples, but train it, or learn the neural network parameters from data, by repeatedly choosing a pair of language samples, scoring each sample individually, and adjusting the model's parameters to make it more likely that the sample originally produced at a later time receives a higher score.

Our neural model of child language development can be thought of as being composed of two modules, which together can assign a score to a language sample containing a certain number of utterances from a child. The first module consists primarily of an LSTM language model, or more precisely a Bidirectional LSTM (BiLSTM) encoder (Graves, 2012), which is used to encode utterances into a vector representation. Given a language sample composed of a certain number of utterances, the LSTM language model encodes each utterance simply by processing the utterance one word at a time. Recall that every utterance ends with a special EOS token. It is the activation of the topmost hidden layer of our model at the last time step, which corresponds to having the EOS token as input, that we use as (half of) the representation of the sentence. This specific representation is chosen because it is the result of the model having processed all of the words in the utterance, and the recurrent nature of the model makes it possible, in principle, for information about the entire utterance to be captured at this last time step. A common practice when encoding strings with an LSTM network is to repeat the process on the reversed string with separate parameters, resulting in a bidirectional model. The string is then encoded forwards and backwards. In the forward pass, the hidden representation for the EOS token is used as half of the representation for the sentence. In the backward pass, the hidden representation for the BOS token gives us the other half of the representation for the sentence. These two halves are simply concatenated. Once representations for individual utterances are computed, a single representation for the entire language sample composed of these individual utterances is simply the average of the representations of the individual utterances. Each utterance representation is a vector, and the representation for the entire language sample is taken to be the average vector of all utterance vectors in the sample.

**Figure 4:**
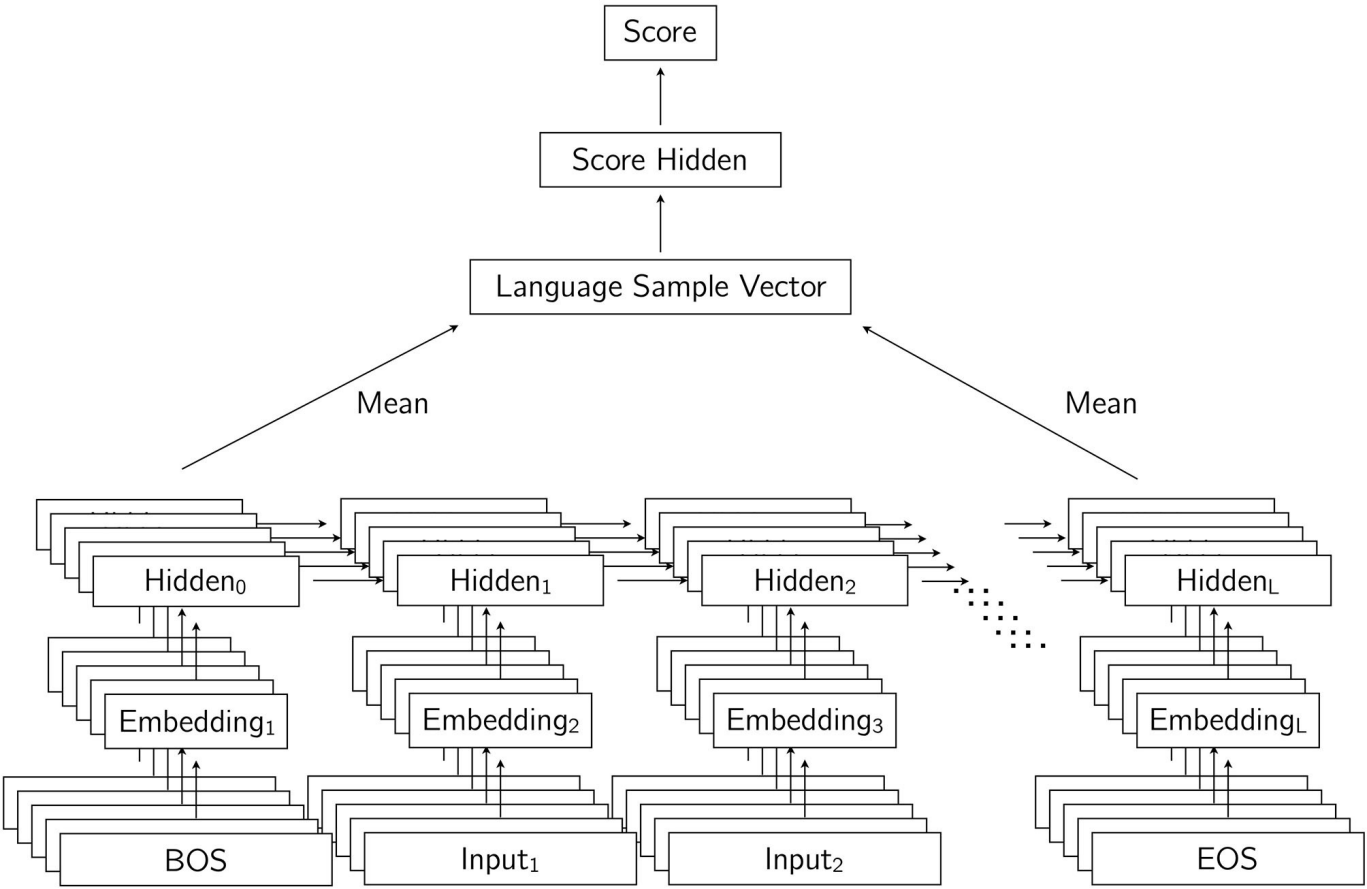
Our model for encoding and scoring language samples composed of utterances. Each utterance is encoded by a Bidirectional LSTM network. Utterance representations consisting of the concatenations of the first and last tokens are averaged into a vector that represents the entire language sample. From this language sample vector, a score is computed for the entire language sample.

Once a vector representation for a language sample is computed using the encoder module containing the BiLSTM language model, the second module of the model derives a numerical score from the representation of the language sample. The scores assigned to language samples from a single child are meant to increase according to the chronological order of the language samples. In other words, the score corresponding to a set of utterances produced by a child of age 3;00 should be greater than the score assigned to a set of utterances produced by the same child at age 2;06. The module that assigns the score to a language sample given its representation consists of a feedforward network that has one hidden layer and a single output unit. The input to this module is the representation obtained with the first module, and the activation of the output unit is the score for the sample. Figure 4 shows our model, with input consisting of several utterances, which are each encoded to create a representation for the entire set of utterances (labeled as Language Sample Vector), from which a score is computed.

With the two modules that together encode a list of utterances and produce a language development score, the remaining questions are how to make the encoder module focus on how the grammar of utterances change over the course of language development, and how to make the score produced by the second module track language development based on what the first module encodes. These two questions are addressed jointly through end-to-end training of the model. An important distinction between our utterance encoder and a typical neural language model trained as described above using a word prediction objective is that our encoder is trained using the language sample sorting task directly. When a typical language model is trained without a specific task as an objective, it learns from its training strings what it needs for its word prediction task. The same network architecture can also be trained on tasks that are not word prediction tasks. In the word prediction case, the error signal that is used to adjust the weights of the network comes directly from the model predicting a different word from what was observed in a specific position in a training string. In our case, an error signal is obtained when the language model has been used to encode the utterances in two distinct language samples, the scorer module assigns scores for these two language samples, and the sorted order of the scores does not correspond to the chronological order of the samples. In such a case, the error is propagated through the entire network so that weights can be updated in a way that is specific to the task.

Training the model requires longitudinal language data from multiple children. For each child, 100-utterance language samples are organized chronologically. Parameters (network weights) for both modules are initialized randomly. During training, the model is presented with data from each child 20 times, and each time a maximum of 100 samples are chosen randomly from the samples from that child. Every language sample is encoded with the first module and scored with the second module. Within the set of 100 randomly chosen samples, every sample is paired with every other sample to create 100 × 99 training pairs, each composed of two samples. In each sample, the chronological order is known. The scores for the two samples are then compared. The model's training objective is to make sure chronologically later samples have higher scores than earlier samples. The number of times data for each child is presented (20), and the number of samples chosen randomly for each child (100) are meta-parameters of the model. The model's meta-parameters and meta-parameter tuning process are described in the next section.

The model is trained end-to-end, with a pair of language samples being provided to the model, each sample being scored and compared, and parameters across the entire model being adjusted in response to errors. This means that the two modules are trained together and influence each other. With weight updates (parameter learning) in neural networks being error-driven, each time an incorrect prediction is made (i.e., the model fails to predict the chronological order the samples), the error is propagated from the ultimate prediction, down to the representation of the average of the utterances in each sample, down to the representations of each individual utterance produced by the language model encoder, and all of the parameters in the entire model are updated to make the correct prediction more likely. Over the process of training the entire model, the language model learns to prefer encodings of the utterances that will make the chronological ordering task more accurate. As a result, the encoder learns to model language development by focusing on the differences in the representations of the two language samples from different times. Because the training material consists of data from multiple children, the model prefers patterns that apply generally, and not to individual children.

Intuitively, one can imagine a very patient intelligent entity with limited memory and no knowledge of grammar, but high sensitivity to details, looking at two sets of utterances produced by the same child several months apart. This entity knows what set of utterances was produced later, and starts to look for patterns that could be used to determine the chronological order of the samples. Initially, the presence of individual words or sequences of words might seem promising, but when presented with a long list of pairs of languages samples, this entity notices that certain structural patterns are more predictive of chronological order. If it is really structural patterns that are most predictive of order of the samples, over many passes over many pairs of samples, the entity will learn a list of what patterns to look for, and how to weigh these patterns against other patterns. This list might end up being similar in many ways to the list of structures used in metrics like IPSyn. This is approximately what motivates our model.

Finally, to prevent the model from picking up on differences in the topics discussed at different ages or the differences in vocabulary, we use the morphosyntactic tags (MacWhinney, 2000) from US English CHILDES transcripts instead of the surface word forms as the tokens in our model. These tags differentiate between parts-of-speech such as nouns, verbs, adjectives, adverbs, prepositions, pronouns, etc. Experiments using the observable surface forms (the words themselves) produced very similar results.

### Implementation Details

The encoder in our model is a BiLSTM with a 50-unit embedding layer and seven hidden layers, each with 200 units for each direction (forward and backward). To encode a language sample, the BiLSTM encoder produces encodings for each utterance as a vector of 400 dimensions resulting from the concatenation of the topmost hidden layer for each direction at the last time step (i.e., the 200-dimensional vector obtained after processing the EOS token in the forward direction, and the 200-dimensional vector obtained after processing the BOS token in the backward direction). We chose the size of the language samples to be 100 utterances, motivated partly by the size of the language samples used to computer IPSyn scores.

The scoring module is a feedforward network with one hidden layers of 200 units, and a single output unit. It takes the representation of a language sample as a vector of 400 dimensions and produces a real-valued score. The ranking task used to train the network involves encoding and scoring two language samples, and comparing the resulting scores for each language sample.

The network is trained end-to-end for 20 epochs, and the data for each child in the training dataset is observed once per epoch. The number of epochs was chosen by observing performance on a small part of the available training data that was used as held out or validation data after each epoch. Changes in results after 15 epochs as small, and no significant improvement was observed during meta-parameter tuning after 20 epochs. The number of hidden layers, hidden units and embedding dimension was similarly tuned by using a small held out portion of the training set as a validation set. The meta-parameters of the model were not tuned exhaustively, and it may not be the optimal values. Parameters of the model were optimized using the Adam optimizer (Kingma and Ba, 2015) using a learning rate of 1e-05 and the margin ranking loss function:

$$\begin{array}{l} loss\left(x_{A},\;x_{B},\;y\right)=\max\left(0,\;-y\left(x_{A}-x_{B}\right)\right) \end{array}$$

Here, *x*_*A*_ is the score for language sample *A*, *x*_*B*_ is the score for language sample *B*, and *y* is +1 if *A* comes before *B* chronologically and −1 if *B* comes before A chronologically. When the model's predictions for the scores of the two samples order the samples correctly, the loss is zero. Otherwise, the loss is greater than one, and the value is used in parameter updates to reduce loss.

### Data

To train and evaluate our model, we used data from the CHILDES Database. Training our model requires longitudinal data from multiple children, and we included in our dataset utterances from corpora that contained transcripts collected from the same child at least 6 months apart. Additionally, we included only corpora from which we could extract at least 75 language samples containing 100 complete utterances not including repetitions, and for which we could determine the age of the child in months. Having a certain number of language samples per child ensures that the data will be useful to the model during training, and that we reduce the amount of noise in our evaluation. While it is possible that corpora with fewer than 75 samples would also be useful, we found there were a sufficient number of corpora that fit our criteria. Data from other children that did not fit our criteria was used as development data and in the process of meta-parameter tuning. The corpora and 16 children included in the final dataset are:

- Braunwald: Laura
- Brown: Adam, Eve, Sarah
- Clark: Shem
- Demetras1: Trevor
- Kuczaj: Abe
- MacWhinney: Ross
- Sachs: Naomi
- Snow: Nathaniel
- Suppes: Nina
- Weist: Benjamin, Emily, Jillian, Matt, Roman.

The transcripts for each child were split into samples of 100 utterances each, and the child age corresponding to each sample was recorded to determine the reference ordering during training and evaluation. From each transcript in CHAT format (MacWhinney, 2000), we used the %mor line, containing part-of-speech and morphological analysis for each utterance. To conduct experiments excluding word forms to avoid having our model capture the effect of topic in ordering samples, we simply used the most basic form of each lexical item's tag (e.g. n for nouns, v for verbs, adj for adjectives, etc.), excluding the base form of words and morphological information.

### Experiments

To investigate the extent to which our model can capture information about language development, we implemented our model using PyTorch (http://pytorch.org) and used the dataset described in the previous section for training and evaluation. All computation was performed on a workstation with two 8-core Xeon processors, 256 Gb of RAM and an Nvidia Titan X GPU. We compared the ability of our model to track language development chronologically with how well three baseline metrics perform the same tasks. Our baselines are the Mean Length of Utterance (MLU; Brown, 1973), the Developmental Sentence Score (DSS; Lee and Canter, 1971) and the Index of Productive Syntax (IPSyn; Scarborough, 1990). MLU, DSS and IPSyn scores were obtained for all language samples used in our experiment using the implementations available in the CLAN tools for language analysis (MacWhinney, 2000). These baselines are meant to represent what can be obtained with a straightforward approach that does not require structural analysis of language samples (MLU), and more precise assessment instruments that were designed based on fine-grained language-specific knowledge that require linguistic analysis (DSS and IPSyn).

While scores for MLU, DSS, and IPSyn were obtained simply by running the available tools on each of the language samples, to obtain scores for our model we used our dataset in a leave-one-child-out cross-validation scheme. This means that with a dataset including data for 16 children, we trained 16 different models, each excluding all data from one child. Transcripts for each of the 16 children were then scored using a model that was trained with no data for that specific child. To score transcripts from children outside of our dataset, we would simply train a single model using data for all 16 children in our dataset. Our leave-one-child-out cross-validation allows us to estimate how the model performs on unseen children by each time training with data from 15 children and scoring transcripts from a child excluded from the model.

Unlike in previous work to automate measurement of syntactic development (Sagae et al., 2005; Hassanali et al., 2014; MacWhinney et al., 2020) or to obtain a data-driven approximation to an existing metric (Lubetich and Sagae, 2014), the target for the scores in our model is not simply another value that can be derived for each transcript, such as an IPSyn score or age in months. Since the goal of our model is to track development over time and assign scores that reflect the chronological order of language samples for a child, we evaluate our model and compare it to baselines based on this task directly. For each child, we compute the Spearman rank correlation coefficient between the scores for each language sample and the child's age in whole months corresponding to each language sample. The Spearman coefficient, or Spearman's ρ, ranges from −1 to +1 and reflects the strength of the correlation between two rankings. Our reference ranking is the age in months. A perfect Spearman rank correlation of +1 would indicate that the scores assigned by our model perfectly sort the language samples chronologically. A Spearman rank correlation of zero would indicate that there is no correlation between the order derived from the scores of our model and chronological order. The stronger the correlation, the better suited for tracking language development we consider a metric to be.

## Results

We compute Spearman coefficients for each child between age and MLU, age and DSS scores, age and IPSyn scores, and age and the scores assigned to transcripts by our model. Since we compare these coefficients to each other directly, we obtain a bootstrapped error estimate for each coefficient by resampling the set of transcripts used to compute the Spearman coefficient 10,000 times. Table 1 shows the results obtained for each of the 16 children using MLU, DSS, IPSyn, and our neural network model. For the convenience of having a single value that represents how well each of these metrics correlate with language development over time, we also provide the average values of all children per metric. However, we caution that the meaning of such an average value may not be straightforward to interpret in isolation, and especially across different datasets. Since the set of transcripts for each child contains transcripts from a different range of ages, it is expected that the rank coefficient from some children will be higher than for others. Intuitively, it is easier for any of these metrics to rank two samples 2 years apart than it is to rank two samples 2 months apart. Therefore, these scores are meant to be interpreted in relation to each other. For example, we would not claim that IPSyn scores have an average rank correlation of 0.77 with age, and rather that the average rank correlation is 0.77 for this specific dataset.

**Table 1:** Spearman rank correlation coefficients between age in months and four language development scores for the 16 children in our dataset.

**Corpus: Child**	**MLU**	**DSS**	**IPSyn**	**Our model**
Braunwald: Laura	0.732 ± 0.001	0.794 ± 0.001	0.867 ± 0.001	0.888 ± 0.001
Brown: Adam	0.942 ± 0.000	0.739 ± 0.001	0.906 ± 0.001	0.964 ± 0.000
Brown: Eve	0.976 ± 0.001	0.958 ± 0.000	1.000 ± 0.000	1.000 ± 0.000
Brown: Sarah	0.935 ± 0.000	0.953 ± 0.000	0.966 ± 0.000	0.959 ± 0.000
Clark: Shem	0.842 ± 0.002	0.855 ± 0.001	0.936 ± 0.001	0.889 ± 0.001
Demetras: Trevor	0.618 ± 0.003	0.567 ± 0.002	0.609 ± 0.003	0.727 ± 0.003
Kuczaj: Abe	0.855 ± 0.001	0.804 ± 0.002	0.943 ± 0.001	0.801 ± 0.001
MacWhinney: Ross	0.610 ± 0.002	0.588 ± 0.002	0.458 ± 0.002	0.604 ± 0.002
Sachs: Naomi	0.732 ± 0.002	0.869 ± 0.001	0.933 ± 0.001	0.92 ± 0.001
Snow: Nathaniel	0.190 ± 0.004	0.892 ± 0.001	0.905 ± 0.001	0.881 ± 0.001
Suppes: Nina	0.896 ± 0.001	0.896 ± 0.001	0.896 ± 0.001	0.974 ± 0.001
Weist: Benjamin	0.607 ± 0.004	0.927 ± 0.000	0.964 ± 0.001	1.000 ± 0.000
Weist: Emily	0.336 ± 0.003	0.643 ± 0.002	0.629 ± 0.002	0.432 ± 0.003
Weist: Jillian	0.321 ± 0.005	0.243 ± 0.005	0.126 ± 0.006	0.657 ± 0.003
Weist: Matt	0.685 ± 0.002	0.741 ± 0.001	0.622 ± 0.002	0.713 ± 0.001
Weist: Roman	0.311 ± 0.003	0.735 ± 0.003	0.566 ± 0.002	0.509 ± 0.002
Average	0.662	0.763	0.770	0.807

*The four language development scores include our data-driven approach and three baselines: Mean Length of Utterance (MLU), the Developmental Sentence Score (DSS), and the Index of Productive Syntax (IPSyn)*.

The results in Table 1 show that, while MLU is an effective approach to approximate the level of language development over time across a variety of children, both DSS and IPSyn perform better, as expected. The average Spearman coefficient between age and MLU is 0.662, the lowest correlation between age and a tested metric. The coefficients for DSS and IPSyn are very close, 0.763 and 0.770, respectively. The average Spearman coefficient between age and our model is 0.807. The scores obtained with our model correlate with age to a higher degree than DSS or IPSyn scores do in this dataset, but this is likely due at least in part to the fact that the model was tuned with these transcripts in mind. Although meta-parameter tuning was performed based on results obtained using transcripts from children not used in our evaluation, various factors such as the number of units and layers in the network and the learning rate were influenced by observing the training process itself, even if separate validation transcripts were used. Still, our results indicate that our model, which uses no pre-specified language-specific knowledge and learns its parameters entirely from transcripts, performs on par with metrics designed by experts to capture language-specific phenomena. This is a significant result in that the model derives all of its knowledge of the language and of the task from the training dataset consisting of utterance sets from various children.

In Table 1, we observe that for some children, there is a very strong correlation between age and all of the different scoring approaches we used. For example, among the three children in the Brown (1973) corpus, all rank correlation coefficients are above 0.9, with the single exception of DSS for one of three children (Adam). The rank correlation between MLU and age is strongest for the children in this corpus, perhaps not surprisingly given the role of these data in establishing MLU as an effective metric. On the other hand, the correlation between MLU and age is weakest in the four children in the Weist corpus (Weist and Zevenbergen, 2008). Among the other metrics, only our model outperforms MLU across all four children in this set, although the age correlation of our model for one of the children (Emily, 0.432) is substantially below the age correlation values for DSS (0.643) and IPSyn (0.629).

## Discussion

### Toward Data-Driven Metrics for Language Development

The use of automated methods for computation of fine-grained language development scores that take syntactic structure into account is a promising application of current natural language processing techniques. Despite some success in the application of automatic syntactic analysis to this task (Sagae et al., 2005; Hassanali et al., 2014; Lubetich and Sagae, 2014), these past efforts served more to demonstrate feasibility than to provide practical tools that can be used routinely in a variety of research situations. Roberts et al.'s (2020) recent effort to perform an independent evaluation of an implementation of automatic IPSyn scoring, and the subsequent effort to improve automatic scoring based on that evaluation (MacWhinney et al., 2020) highlight the amount of care and engineering effort required to make reliable automatic scoring widely available. The very small number of languages for which a detailed metric such as IPSyn is available further stresses the scale of the larger task of making resources available for language development research in various languages, allowing for both greater depth of language-specific findings and cross-lingual research. We present a different way to approach this situation through data. While our current goal is not to provide a new metric for English or any specific language, we show that current neural network language modeling is capable of capturing some aspects of the language development process to the extent necessary to track language development in individual children at a level of precision substantially greater than with MLU and comparable to that obtained with a detailed language-specific metric such as IPSyn. Our results can serve as the foundation for data-driven metrics in different languages, requiring only longitudinal data in the form of transcripts. Once the model is trained, it can be used to score a language sample from a new child by first encoding the utterances using the BiLSTM language model, and scoring the resulting using the feed-forward network. Unlike the training process, which requires several passes through a sizable collection of transcripts, scoring new language samples can be done seemingly instantly with a current consumer-grade general-purpose computer. The amount of computation required for scoring a language sample is greater than what would take to obtain the MLU score for the same language sample, but it is comparable to the amount of computation required for automatic IPSyn scoring.

The use of language samples from multiple children during training results in a model that produces scores that are not specific to any one child and are comparable across children. Since training consists of repeated attempts to predict the order of language sample pairs from different children using a single scoring model, these scores can be used to compare the level of development of a child to that of another child, or to a mean value for a group, in a similar way to how MLU or IPSyn scores are used. However, unlike MLU and IPSyn, which operate on known scales defined explicitly, the scores from our data driven model are dependent on the dataset used for training. With the model as described above, there is not even a pre-defined range for the scores produced by the model. In fact, scores from models trained with different datasets may not be directly comparable numerically. To keep the scores of a practical language development metric that uses our approach and a specific dataset within a pre-defined range, a sigmoid function can be applied to the value produced by the scoring module.

The results in Table 1 provide a strong indication that neural network language models trained with longitudinal data can capture structures relevant to the measurement of language development. In addition to adding to the growing body of knowledge related to whether and how neural networks can derive syntactic structure from text alone, our work also points to an area of application of this apparent ability of recurrent networks to model language structure. However, our experiment involved data for only 16 children, and much work still needs to be done toward a usable metric or a set of metrics for various languages. Further validation of our approach through extrinsic methods, such as verifying that previous research results obtained with IPSyn, LARSP, or DSS scores can be replicated with scores obtained from our fully data-driven model, would be needed to examine the potential practical utility of the approach.

One aspect in which metrics such as IPSyn and DSS that hold a considerable advantage over a fully data-driven approach is interpretability of scores. With IPSyn and DSS, scores are tied directly to a known procedure in a way that is fully transparent. Furthermore, subscales can give additional insight through a more detailed view of language development. While neural network models should not be considered uninterpretable, and a growing body of research is dedicated specifically to understanding what neural language models learn (Rogers et al., 2020), this kind of work is still in its infancy, and not yet at a stage that can provide clear information about what specific kinds of information a model such as ours learns from language data.

### The Role of Syntax in Measuring Language Development

Previous research on interpreting what kind of syntactic information is encoded in neural language models and on explicit modeling of syntax with neural networks suggest that our model's ability to track language development over time must be due to our BiLSTM encoder's ability to capture at least some relevant aspects of syntax, and the entire model's ability to capture what structures are expected to appear through the process of language acquisition. Previous work has shown that even with the simple language model objective of word prediction, BiLSTM and related neural network architectures can learn some syntactic structure (Futrell and Levy, 2019; McCoy et al., 2020; Linzen and Baroni, 2021). In discussing structures that are not learned well by recurrent neural networks using the next-word prediction task, Linzen et al. (2016) suggest that even in those cases, the use of other training objectives may result in learning of these structures. In fact, the success of syntactic parsers built on top of BiLSTM encoders with the explicit objective of predicting syntactic structure of input strings (Kiperwasser and Goldberg, 2016) shows convincingly that BiLSTMs are capable of capturing syntactic structure, especially given an appropriate objective. Beyond investigations into whether recurrent neural networks encode syntax, the apparent fluency of LSTM language models for language generation, including in machine translation (Sutskever et al., 2014), suggest these models learn a fair amount of syntax, since fluent generation would be unlikely without it. Still, the question remains if our model learns to score language development based on syntax or other more superficial features of the utterance strings, such as length.

While it is a safe assumption that our model does learn to leverage utterance length in scoring, since it contains information relevant to the task, as shown by MLU, it is unlikely that the performance of the model can be attributed to superficial string characteristics alone. The levels of rank correlation with age obtained with scores produced by our model, compared to those obtained with MLU, with DSS and with IPSyn further suggest that our model captures syntactic development. Given the similarity of the correlation coefficients obtained with our model and with IPSyn and the extent to which IPSyn scores are based on syntactic structures, it is reasonable to expect that our model's success is due to its modeling of syntax. Initial experiments with the original word forms in the utterances produced very similar results as the ones presented, but it was not clear if the model learned what changes in the grammar as language development progresses, or what children tend to talk about at different ages. To isolate the effect of syntactic structure, we used only plain part-of-speech tags to represent the words in the utterance strings, completely removing any information about topic. This makes it likely that the model does in fact rely on syntactic structure, especially since our neural language model encoder is trained not with the word prediction objective, but the language sample ordering objective.

To examine the extent to which our model relies on utterance length, we performed an ablation experiment where we remove structure from the utterances used to train our model, but leave utterance length intact. This is done simply by replacing each token with the same arbitrary symbol, so that each utterance is as long as before, but it is made of the same symbol (word) repeated over and over. This ablated model that only considers length is trained and evaluated in the same way as our model. Recall that the levels of age correction for MLU and our model are 0.662 and 0.807, respectively (Table 1). The correlation coefficient for age and our length model is 0.711, putting it closer to MLU than to our full model. Although this ablated model scores language samples based on length, like MLU, the advantage it has over MLU is that it can consider the distribution of lengths of the utterances in the sample, and not solely the mean. For each child in our dataset, the coefficients for MLU and for the length model were similar, with the exception of Naomi from the Sachs corpus (0.732 with MLU vs. 0.910 with the length model) and Nathaniel from the Snow corpus (0.190 with MLU vs. 0.476 with the length model).

Although it is clear that our model captures more than just utterance length, the question of what else it captures remains. To examine our conjecture that the model identifies syntactic information in utterances, we performed an additional experiment using our fully trained model. Recall that our model is composed of two modules: a BiLSTM network that encodes utterances, and a feed-forward network that produces a score based on the encoding produced by the BiLSTM network. If the model learns syntactic structure, this information would be present in the BiLSTM network. To test whether our model in fact uses identifiable syntactic structure, we used the syntactic structure annotation available in the American English transcripts in the CHILDES database. Each utterance in these transcripts is accompanied by a syntactic analysis in the form of a dependency structure that represents grammatical relations computed automatically by a data-driven parser (Sagae et al., 2010). Using the same transcripts as in our evaluation of our model, we find the 20 most common syntactic dependency types across all utterances, and try to determine whether our BiLSTM utterance encoder can detect the presence of each of these dependency types in individual utterances. The 20 most common syntactic dependency types in our dataset, ordered more to less common, are: SUBJ (subject), ROOT (main verb), JCT (adjunct), DET (determiner), OBJ (object), AUX (auxiliary), POBJ (object of a preposition), PRED (predicate nominal), LINK (complementizer, relativizer, or subordinate conjunction), MOD (non-clausal nominal modifier), COMP (clausal complement), COM (communicator), INF (infinitival *to*), NEG (negation), QUANT (quantifier), NJCT (nominal adjunct), COORD (coordination), CONJ (conjunction), CMOD (clausal nominal modifier), and XMOD (non-finite nominal modifier). Explanations for each of these dependency types in the context of syntactic analysis of CHILDES transcripts can be found at https://talkbank.org/manuals/MOR.html.

For each of these syntactic dependency types, we construct a dataset containing an equal number of utterances where the corresponding grammatical relation appears and utterances where the corresponding grammatical relation does not appear. We then encode each of these utterances using our BiLSTM utterance encoder to obtain a vector representation for the utterance, as described in section A Neural Network Model of Child Language Development. This vector is the concatenation of the encodings of the beginning of sentence token (BOS) and the end of sentence token (EOS). We then train a classifier to detect whether each of these fixed length vectors correspond to an utterance where the grammatical relation in question appears or does not appear. For example, we take an equal number of utterances containing a CMOD dependency relation (approximately, a relative clause) and not containing a CMOD relation, and train a binary classifier (in this case, a feed-forward network with 50 hidden units) to predict if the original utterance contains a CMOD relation. These vector encodings do not contain the tokens in the original utterance, so this prediction must be made based on what information from the utterance the model encodes once it is trained. Over the course of training of the model, these vector encodings are expected to capture the information necessary for ordering utterances chronologically. If a specific syntactic structure, such as a relative clause represented by the syntactic dependency type CMOD, is useful to the model in the ordering task, we expect to be able to detect whether or not the utterance contains a relative clause from the vector alone. We use an equal number of utterances containing and not containing each dependency type so that identification of dependency types cannot be made based on frequency information. Finally, we test each classifier on an unseen set of utterances also consisting of an equal number of utterances containing and not containing the dependency type in question.

The accuracy of these classifiers, shown in Table 2, confirm that our model does capture a substantial amount of syntactic information. Since each syntactic dependency type is tested with an equal number of utterances containing and not containing the dependency type, an accuracy of 50% would correspond to no ability to detect the dependency type from the vector encoding of the utterance, while an accuracy of 100% would correspond to perfect ability to detect the dependency type, which would require the presence of the syntactic dependency to be encoded in the vector. Since the syntactic annotation used to train our classifiers experiment is produced automatically, and therefore noisy, it would be unrealistic to expect accuracy of 100%. Each dependency type was identified by its corresponding classifier with accuracy of at least 60%. CJCT (85.9%), ROOT (84.4%), and SUBJ (82.3%) were the dependency types identified with highest accuracy, and MOD (61.3%), QUANT (64.1%), and XMOD (69.2%) were the dependency types identified with lowest accuracy. These results support our expectation that our model encodes syntactic structure.

**Table 2:** Accuracy in detection of the 20 most common syntactic dependency types in our dataset from utterance encodings produced by our model.

**Syntactic dependency type**	**Accuracy (%)**
SUBJ	82.3
ROOT	84.4
JCT	74.1
DET	72.3
OBJ	73.6
AUX	79.2
POBJ	73.9
PRED	72.7
LINK	72.1
MOD	61.3
COMP	78.9
INF	81.8
NEG	75.1
QUANT	64.1
NJCT	74.3
COORD	72.5
CONJ	75.8
CJCT	85.9
CMOD	75.1
XMOD	69.2
AVERAGE	74.9

### Language-Specific and Population-Specific Considerations

Being completely data-driven, we expect our model to be suitable for modeling language development in languages other than English. However, since training our model requires a number of transcripts from the same child over some period of time, application of our method to the vast majority of languages is far from trivial. While the CHILDES Database does contain a limited amount of suitable data for a few languages, no languages have an amount of data that even approaches what is available for English. Although the apparent trade-off between a top-down approach where structures are enumerated by an expert and a bottom-up approach where relevant structures emerge from data may seem to favor the top-down view for the moment, we are experiencing an unprecedented increase in the availability of language data of many different kinds. For many reasons, child language data is not as readily available as many other kinds of language data, but collection of the necessary data to create a model similar to ours in other languages appears to be a feasible, although non-trivial, task. Although concerns about safety and privacy remain, the ability to record, store and share naturally occurring language, and advances in automatic transcription (Gurunath Shivakumar and Georgiou, 2020) make the effort to build the necessary datasets increasingly more manageable. While we are still in a situation where large areas of research are too heavily focused on English, it is our hope that a data-driven approach will create new opportunities for multilingual and cross-lingual research, as has been the case with automatic syntactic parsing (Zeman et al., 2018).

An exciting possibility created by a data-driven, bottom-up modeling approach is that language development can be considered not just from the perspective of different languages, but from the perspective of different populations with different varieties of the same language. Even within the context of American English, one must consider that within the United States alone there are substantial language differences among populations, and the validity of metrics is usually examined for one variety, with applicability to other varieties being the topic of separate studies (Oetting et al., 2010). While MLU values can be interpreted in the context of different populations, this advantage is due to how coarse-grained the metric is. More precise metrics based on inventories of specific structures would need to be adapted based on expertise of the relevant language structures for each population. Given the appropriate datasets, the data-driven view allows for precise, fine-grained scoring relative to a population represented in a specific dataset, without the need for the assumption of a mainstream or standard variant at the expense of other equally valid variants. Although MLU is the most convenient approach for assessment of language development, since it does not require a language-specific scoring scheme like IPSyn does, and it does not require a longitudinal dataset like our data-driven approach does, it is not as precise as the alternatives considered. When considering the application of an approach like IPSyn or our data-driven approach to a new population whose language may not be identical to that of populations used to validate these metrics, one is faced with a typical top-down vs. bottom-up trade-off. If no data is available and data collection is impractical, one might be well-served by turning to language expertise to adapt a metric like IPSyn. When considering the amount of variety in English, and especially going beyond English, this approach may be difficult to scale, and will continue to be difficult to scale. The data issue, on the other hand, is likely to continue to become easier to deal with, based on the trend observed for the past couple of decades. While this brings non-trivial questions about best practices for construction of datasets that represent a language or a specific variant of a language, it is preferable to address these questions imperfectly but explicitly than to leave them unacknowledged, hiding the potential for inequity in research results.

## Conclusion

Advances in natural language processing, and specifically in language modeling using neural network approaches, create exciting opportunities for modeling language development, including how grammatical structures develop over time. Motivated by recent work that shows that recurrent neural networks learn some aspects of syntactic structure when given appropriate training objectives (Kiperwasser and Goldberg, 2016; Futrell and Levy, 2019; Linzen and Baroni, 2021) and by previous work on data-driven measurement of syntactic development (Lubetich and Sagae, 2014), we show that a model composed of a Bidirectional LSTM to encode language samples and a feedforward network to score encoded samples can be as effective at producing language development scores that can track child language development over time as detailed language-specific metrics designed by experts, such as the Index of Productive Syntax (Scarborough, 1990). Although our goal is not to create a new metric for language development in English, and several issues remain unaddressed before our work can be leveraged into metrics that can be used in practice, our work is significant in that it shows that recurrent neural networks, without any pre-specified knowledge about language beyond the inductive bias inherent in their architecture, can learn the child language acquisition process to the extent necessary to track language development in sets of transcripts as accurately as established metrics. We support our claim that our model learns syntactic structure by showing that it outperforms a baseline based on Mean Length of Utterance, and by removing all semantic information from transcripts to prevent the model from leveraging topic information and other cues.

In addition to demonstrating how neural language models can capture the language development process successfully, we hope that our work will serve as the basis for future work on modeling and measuring language development that, due to its bottom-up data-driven nature, will focus on a wider variety of languages and language varieties, creating the possibility for new language-specific and cross-lingual research on child language and development of syntax.

## Data Availability Statement

Publicly available datasets were analyzed in this study. This data can be found here: http://childes.talkbank.org.

## Author Contributions

The author confirms being the sole contributor of this work and has approved it for publication.

## Conflict of Interest

The author declares that the research was conducted in the absence of any commercial or financial relationships that could be construed as a potential conflict of interest.
